# Successful management of chronic migraine through manual therapy. A case report

**DOI:** 10.25122/jml-2023-0222

**Published:** 2023-06

**Authors:** Judit Mihaiu, Florian Bodog, Aurel Mohan, Georgiana Vieriu

**Affiliations:** 1 Doctoral School of Biomedical Sciences, University of Oradea, Oradea, Romania; 2 Faculty of Medicine and Pharmacy, University of Oradea, Oradea, Romania

**Keywords:** migraine, pain, tenderness, manual therapy

## Abstract

Migraine is the second most debilitating condition affecting a significant portion of the general population, particularly young adults under 50. Despite extensive research, the etiology of migraine remains diverse and often multifactorial, with limited clarity on the specific underlying pathogenic processes. Consequently, the treatment of migraine lacks a mechanism-based approach. We present the case of a young female patient suffering for 8 years of chronic migraine and tension-type headaches. The manual therapy techniques successfully released the muscular tensions, considerably improving her quality of life in terms of pain and emotional well-being.

## INTRODUCTION

Migraine is a complex and diverse nervous system disorder known for its high prevalence and significant impact on individuals' quality of life [1]. It ranks as the second most debilitating condition in the general population and the leading cause of disability among young adults under 50 [2-4]. Over the years, significant progress has been made in understanding migraine, leading to adjustments in the diagnostic criteria based on new findings. While the exact pathogenesis of migraine remains incompletely understood, it is widely believed to involve the activation of the trigeminovascular system, both peripherally and centrally [5]. The underlying neurophysiological substrate of migraine aura seems to be a cortical spreading depression [6]. No genetic alterations have been identified yet, although the migraine is commonly familial.

Migraine is characterized by recurrent headache attacks lasting anywhere from 4 to 72 hours. Typically, these attacks are characterized by pulsating pain located unilaterally (in children and adolescents, it may be bilateral), having a moderate to severe intensity aggravated by routine physical activity (even walking or climbing stairs), and usually associated with nausea and/or photo- and phonophobia [7]. In one-third of individuals with migraine, headache is sometimes or always preceded or accompanied by transient neurological disturbances, referred to as migraine aura.

A migraine attack can be divided into four phases based on their temporal relationship to the headache: the premonitory phase, which precedes the headache and is characterized by nonspecific symptoms such as yawning, mood changes, light sensitivity, increased urination, neck pain, and difficulties with attention and concentration; the aura phase, which immediately precedes or occurs concurrently with the headache; the headache phase itself; and the postdrome phase, which occurs after the resolution of the headache [1]. Furthermore, some affected persons develop chronic migraines with persistent attacks.

Diagnosing migraines relies on the recognition of a recurring headache pattern, along with associated symptoms, by a healthcare professional, usually a neurologist. Accurate diagnosis follows the guidelines outlined in the International Classification of Headache Disorders (ICHD-3) [7] and mentioned above. Migraine treatment is not mechanism-based since the underlying specific pathogenic processes are not clear. It typically involves the use of acute and preventive medications, as well as various non-pharmacological therapies, such as Botox. Although its mechanism of action is not well documented, studies have demonstrated that injecting specific sites on the head and neck significantly benefits chronic migraine patients [8].

## CASE PRESENTATION

In November 2021, a 26-year-old Caucasian female patient presented at the Physiotherapy Private Practice Office with a confirmed diagnosis of migraine based on her symptoms and non-contrast-enhanced MR angiography.

### Assessment

She had been experiencing migraine attacks twice a month and tension-type headaches once a week for over 8 years. The patient was able to differentiate between the two types of headaches primarily due to the presence of aura.

#### Subjective

The patient had a history of daily migraines and tension-type headaches lasting more than 8 years. She denied any history of injury or trauma and had no smoking or alcohol consumption habits. The migraine pain was predominantly in the left temporal, retro-ocular, suboccipital, and frontal (glabella) areas. On a 1-to-10 pain scale, the initial pain intensity was reported as 10. Additional symptoms included nausea, dizziness, numbness in the forearms, hands, left half of the face, mouth, eye floaters, photo- and phonophobia, and bruxism. The patient identified stress, hunger, and cold environments as triggers for her headaches, while light, noise, smells, and certain sounds aggravated her symptoms. She also reported poor sleep quality. The patient had previously tried various prescription and over-the-counter medications, with only Sumatriptan providing effective relief. Additionally, she tried alternative therapies such as laser therapy (at the age of 20), Bowen sessions (twice a week for 3 months), and acupuncture (twice a week for 6 months). She had also undergone Botox injections twice, six months apart, at the age of 20. After the first injection, the frequency of migraines decreased from around 15 days a month to 10 days/month. Following the second injection, the frequency stabilized at 5-7 days a month, consisting of two migraine attacks and a tension-type headache once a week. There were no significant past medical history findings, but a maternal aunt also experienced migraine attacks.

#### Objective

**Figure 1:**
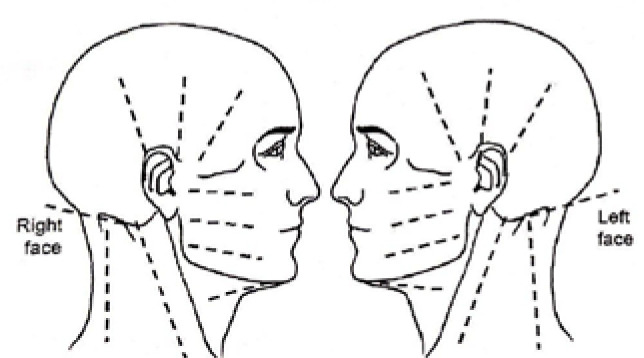
Assessment lines of the lateral face, head, and neck

The assessment methods and instruments were observation, the patient’s report, a questionnaire, and specific tests, all related to the ICHD-3. The questionnaire focused on the location and the characteristics of the pain, the frequency, periodicity, duration, and evolution of the headache episodes, the triggers of the headache episodes, known factors that alleviate or worsen the pain, other symptoms that occur before, during and after a headache episode. The specific tests comprised palpation of muscle groups in the head, face, and neck area (Figure 1) and shoulder areas, as well as a postural analysis. We assessed the occipitofrontalis (both frontal and occipital portions), epicranial (galea) aponeurosis, temporalis, masseter, pterygoid, digastric, upper trapezius, splenius, suboccipital and sternocleidomastoid muscles. We found pain and tenderness in the temporalis, frontal portion, masseter, and suboccipital muscles. Differential diagnosis ruled out cervicogenic headache, as the pain radiated in an anterior-posterior direction, consistent with a diagnosis of migraine [9].

The postural analysis focused mainly on the head, neck, and thorax positions, viewed anteriorly, posteriorly, and from the side, in standing and sitting positions. We found a slight inclination of the head to the left. The shoulders were symmetric, and the thoracic spine showed no sign of abnormal curvature. The methods applied were visual observation, photography, and plumbline.

The Visual Analog Scale was used to evaluate the intensity of the pain. Due to her long history of pain, she was used to monitoring it. Even though the patient did not report pain during the initial assessment, the average intensity of pain experienced in the last month was rated 7 out of 10 on the pain scale. The same scale was used during the following months to monitor the progress.

The Headache Impact Test (HIT-6^TM^) measures the impact of the headache (any of them) on everyday life and activities with good reliability (Cronbach’s α: 0.75–0.92) [10]. The HIT-6 score was 67 (of the maximum of 78). The Migraine Disability Assessment (MIDAS) test, which assesses the impact of migraine on daily life and work (Cronbach’s α of 0.83), indicated a score of 14, corresponding to MIDAS grade III, moderate disability [11]. Emotional symptoms, including anxiety and stress, were also present and contributed to worsened pain and reduced quality of life between attacks. Additionally, the patient developed awake and sleep bruxism, causing increased pain and fatigue in the masseter muscles.

## TREATMENT

The patient maintained a headache diary throughout treatment to monitor her symptoms and encourage self-awareness (Table 1). The diary aimed to provide additional data for the physiotherapist, allowing the patient to track changes in her condition in real time. This diary also had the added benefit of providing emotional support to the patient, helping to alleviate the emotional burden she had experienced due to years of persistent pain.

**Table 1:** The headache diary – first migraine episode after the initial assessment

Date	Time	What were you doing when it began	How long it lasted	Symptoms experienced	Medicines taken (if any)	Other healing methods (if any)	Level of pain (from 1 to 10)
							

The patient attended manual therapy sessions twice a week at the physiotherapist's office, each lasting 45 minutes. The sessions comprised craniocervical muscle exercises, suboccipital inhibition, cervical spine mobilization [12], trigger point treatment, soft tissue mobilization, elongation, muscle release, stretching, and head and neck massage. Additionally, the patient performed a daily 10-minute at-home exercise session to improve posture and flexibility. The primary treatment goals were to reduce the intensity and frequency of pain, alleviate accompanying symptoms, decrease medication intake, and improve the patient's quality of life and work efficiency.

### Follow-up and outcomes

After 7 therapy sessions, the patient reported improved sleep quality, decreased pain intensity from 7 to 5, and reduced medication usage. The headache diary was very helpful in documenting the progress made during the treatment period. After 3 months (14 weeks, 28 sessions of therapy), an intermediary assessment was performed, and the results correlated with the headache diary.

**Figure 2:**
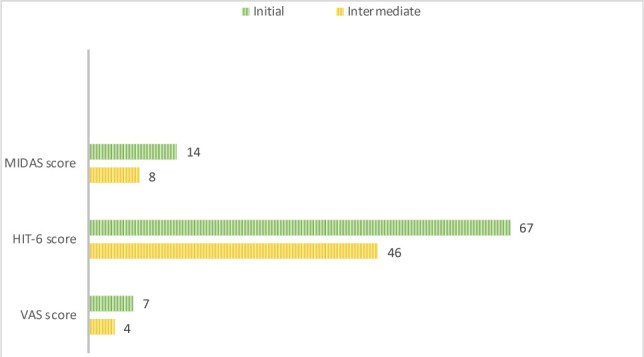
Comparative results between the initial and intermediate assessments

The pain and tenderness in the temporalis, the frontal area of the occipitofrontalis, and masseter muscles decreased significantly and disappeared in the suboccipital muscles. The patient no longer exhibited a head inclination to the left. The VAS score decreased to 4 (the average of the last 4 migraine attacks). The patient did not report any tension-type headaches in the last four weeks. The HIT-6 score was 46, and the MIDAS score was 8 (mild disability, MIDAS grade II) (Figure 2).

**Figure 3:**
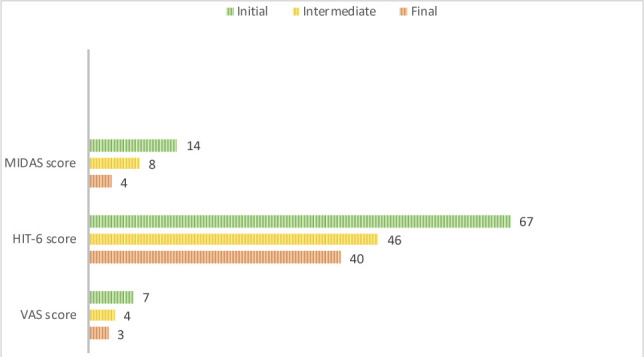
Comparative results between the initial, intermediate, and final assessments

The therapy continued with one weekly session lasting 45 minutes. After 6 months from the initial assessment and 3 months from the interim assessment, a final assessment was conducted. The pain and tenderness of the muscles disappeared, the VAS score was at an average of 2-3, and the tension-type headache was still absent. The HIT-6 score decreased to 40, and the MIDAS score to 5 (grade I, little disability) (Figure 3). Migraine episodes occurred less frequently, with two episodes in the past three months, totaling four days, and minimal use of medication (seven tablets in the two episodes).

The patient reported an increase in quality of life and improved work performance. Currently, she attends weekly sessions to maintain the achieved results, and the headache diary is no longer necessary.

## DISCUSSION

Due to the unclear pathogenesis and lack of targeted treatment options for migraine, it is challenging to provide an exact prognosis for outcomes in most cases. In the case of this young patient, she experienced a prolonged period of severe pain that significantly impacted her quality of life, increasing stress levels (which itself acted as a trigger for her migraine attacks), anxiety, and feelings of hopelessness. Understanding the various contributing factors and their impact on headaches is crucial to effectively address and alleviate the pain.

Through manual therapy, we were able to release a significant amount of tension in the muscles surrounding the skull (occipital, frontal, and temporal muscles), contributing to her pain. While this approach provides relief for migraines exacerbated by associated muscle stiffness, it should be noted that it primarily addresses compensatory measures aimed at reducing muscular adaptations that exacerbate pain [13]. Given the risk-free nature of manual therapy, it is worth considering as a treatment option, particularly since migraines are highly disabling with limited evidence-based interventions available.

## CONCLUSION

The results obtained from the three assessments conducted over six months demonstrate the benefits of manual therapy in managing chronic migraine and tension-type headaches in the absence of other comorbidities. Although ongoing periodic assessments are not considered necessary, the patient continued attending sessions to maintain the achieved results.
